# *Kingella kingae* Virulence Factors and Insights into Pathogenicity

**DOI:** 10.3390/microorganisms10050997

**Published:** 2022-05-10

**Authors:** Eric A. Porsch

**Affiliations:** Department of Pediatrics, Children’s Hospital of Philadelphia, Philadelphia, PA 19104, USA; porsche@chop.edu

**Keywords:** *Kingella kingae*, virulence factors, pathogenesis, type IV pili, toxin, capsule, adherence, invasion

## Abstract

The emergence of *Kingella kingae* as an important etiology of pediatric osteoarticular infections over the past three decades has led to significant research efforts focused on understanding the pathogenicity of this fastidious Gram-negative bacterium. This work has uncovered multiple virulence factors that likely play key roles in the ability of the organism to colonize the upper respiratory tract, breach the epithelial barrier, and disseminate to distal sites of infection. Herein the current body of knowledge about *K. kingae* virulence factors is reviewed in the context of *K. kingae* disease pathogenesis. The work summarized here has identified multiple targets for therapeutic intervention as well as potential vaccine antigens.

## 1. Introduction

The pathogenesis of *K. kingae* disease begins with colonization of the oropharynx, its invasion of the bloodstream, dissemination via the hematogenous route, and finally invasion of distal sites of infection. Over the past two decades, there was considerable investigation into the bacterial factors and mechanisms that promote, modulate, and regulate several steps in this pathogenic process. Our current understanding of these factors and their potential roles in the development of invasive *K. kingae* disease are reviewed and discussed below. By gaining a more thorough understanding of the virulence factors involved in *K. kingae* pathogenicity, there is the potential to uncover novel targets for the treatment or prevention of *K. kingae* colonization and disease.

## 2. Adherence to Host Respiratory Epithelium

### 2.1. Type IV Pili

Early morphological examination of *K. kingae* revealed the presence of long surface fibers [1,2]. Kehl-Fie et al. demonstrated that these fibers were type IV pili and that presence of the fibers on the bacterial surface was essential for high-level adherence to multiple cell types, including epithelial cells and synovial cells [3]. Further examination of *K. kingae* clinical isolates revealed that piliation in this species is highly variable and correlates with colony morphology. Strains that express high levels of type IV pilus fibers produce a spreading/corroding colony type, strains that express low levels of fibers produce a non-spreading/non-corroding colony type, and strains that express no fibers produce a domed colony type [4]. In this collection of clinical isolates, presence of type IV pili, regardless of piliation level, was associated with human epithelial cell adherence [4]. Expression of the primary structural protein that composes the type IV pilus fiber (major pilin, PilA1) was found to be regulated by the PilS/PilR two-component regulatory system and the alternative σ factor σ54 [5]. In prototype strain 269–492, mutation of *pilR* resulted in ablation of piliation, while mutation of *pilS* resulted in a reduction in piliation, suggesting that the response regulator PilR retains a basal level of *pilA1* transcriptional activation potential in the absence of the signal kinase PilS [5].

Two pilus-associated proteins called PilC1 and PilC2 were found to be critical for type IV pilus-mediated functions. Expression of PilC1 or PilC2 alone was shown to be essential for surface piliation and for the host cell adherence phenotype [3]. Mutants lacking both PilC1 and PilC2 are non-adherent and have a severe defect in surface piliation level [3,6,7]. Members of the PilC family of proteins in other bacteria were shown to be multifunctional, playing critical roles in type IV pilus biogenesis and adherence. Interestingly, PilC1 and PilC2 are highly sequence divergent, only sharing 7% identity and 16% similarity overall [3]. Examination of their amino acid sequences revealed a predicted 9-amino acid calcium-binding (Ca-binding) site in the C-terminal region of PilC1 and a 12-amino acid Ca-binding site in the C-terminal region of PilC2. In contrast to what was observed for the PilC-like protein in *Pseudomonas aeruginosa* (called PilY1) [8], elimination of Ca-binding in PilC1 and PilC2 via a mutagenesis approach had no effect on surface piliation [6]. However, Ca-binding by the PilC1 site was shown to be essential for type IV pilus-mediated adherence and a form of type IV pilus-mediated surface motility called twitching motility, while elimination of Ca-binding in PilC2 only had a modest effect on twitching motility and no effect on adherence [6].

Given that expression of at least one PilC protein is essential for both type IV pilus biogenesis and type IV pilus-mediated adherence, the hypothesis that both proteins are pilus biogenesis factors as well as direct adhesins was explored. Recombinant PilC1 and PilC2 were expressed, purified, and examined in binding assays with human epithelial cells [7]. Both proteins were shown to bind to cells in a specific manner, and the binding activity was localized to the N-terminal region of the proteins. Interestingly, expression of truncated PilC1 or PilC2 fragments in *K. kingae* (in the absence of the other PilC protein) revealed that the C-terminal region of each protein was able to promote pilus biogenesis but not adherence [7]. These findings identified *K. kingae* PilC1 and PilC2 proteins as multifunctional proteins involved in a variety of type IV pilus-mediated phenotypes.

### 2.2. Kingella NhhA Homolog (Knh)

Bioinformatic analysis of the *K. kingae* genome aimed at identifying putative surface localized adhesive proteins uncovered a large open reading frame (ORF) with sequence homology to the *Neisseria meningitidis* NhhA trimeric autotransporter adhesin and was named the *Kingella* NhhA homolog (Knh). The predicted C-terminal β-barrel domain was demonstrated to trimerize, confirming that Knh is a trimeric autotransporter [9]. Deletion of *knh* resulted in a ~50% reduction in *K. kingae* adherence to epithelial cells [9]. Further analysis revealed that Knh adhesive activity was dependent on post-translational addition of glucose residues by an unconventional N-linking glycosyltransferase [10]. Examination of *K. kingae* type IV pilus- versus Knh-mediated adherence under dynamic flow sheer stress conditions revealed that Knh mediates stronger adherence than type IV pili [11].

### 2.3. Mechanism of Adherence

It was noted that *K. kingae* strains lacking type IV pili but still expressing Knh are non-adherent [3,9], suggesting that there is another surface factor that blocks Knh-mediated adherence, and that type IV pili are necessary to overcome the Knh inhibitory factor. In considering potential factors that could inhibit Knh-mediated adherence, the presence of a capsular polysaccharide was investigated. Using a combination of genomic analyses, mild acid surface treatment in combination with Alcian blue staining, and cationic ferritin-staining thin section transmission electron microscopy (TEM), *K. kingae* was shown to elaborate a polysaccharide capsule on its surface (discussed in more detail below) [9]. Elimination of the capsule from the surface of the bacterium through disruption of the *ctrABCD* capsule export operon restored Knh-mediated adherence in a *pilA1* mutant non-piliated background, demonstrating that the capsule is responsible for masking Knh adhesive activity. It was further demonstrated that in a wild-type organism, type IV pili are able to overcome the inhibitory influence of the capsule on Knh-mediated adherence through PilT-mediate pilus retraction [9]. As Knh is a surface protein and is likely immunogenic, it is possible that the capsule plays a role in immune evasion by the masking of Knh and preventing antibody binding.

These data enabled the formation of a model of *K. kingae* adherence to host epithelial cells that involves a three-step process: (1) Type IV pili establish an initial interaction with the host cell; (2) the adhered pili retract pulling the bacterium into close contact with the host cell causing displacement of the capsular polysaccharide; and (3) the displaced capsule allows Knh to access its host cell receptor to mediate full-level adherence [9]. This model is supported by additional evidence showing that the capsule on the surface of *K. kingae* extends beyond the length of Knh and that bacteria interact more closely with host cells when the type IV pili are retraction proficient [11].

## 3. Breach of the Respiratory Epithelial Barrier

### 3.1. RTX Toxin

Once *K. kingae* has colonized the respiratory epithelium of the oropharynx it must next breach the epithelial barrier and enter the bloodstream to disseminate. In early studies of in vitro interactions between *K. kingae* and host cells, it was noted that cell monolayers were rapidly disrupted resulting in cell lifting and cell death [12]. Using a transposon mutagenesis screen, it was shown that *K. kingae* produces a broadly active cytotoxin called RtxA in the repeats-in-toxin (RTX) family [12]. The toxin displays hemolysis against erythrocytes and is cytotoxic to epithelial cells, synovial cells, fibroblasts, osteoblasts, and especially leukocytes [12,13]. In a juvenile rat model of invasive *K. kingae* disease, the RtxA toxin was shown to be critical for the ability of the bacteria to produce morbidity and mortality [13], highlighting its important role in an in vivo system that involves invasion of the bloodstream. Of note, significant pathologies were observed in the spleen, bone marrow, and thymus of animals infected with a strain expressing RtxA compared to an isogenic *rtxA* mutant [13].

Expression, activation, and secretion of the RtxA toxin requires five genes, *rtxBDCAtolC*. The *rtxB* and *rtxD* genes encode an ABC transporter system that, along with the *tolC* gene product, constitute a complete type I secretion system for toxin secretion. The hemolytic and cytotoxic activity of RtxA is dependent on activation by RtxC, which acylates two lysine residues of RtxA. Interestingly, there are two different genomic arrangements of the *rtx* genes in *K. kingae*. All strains examined to date appear to split the *rtx* genes across two separate loci, with some strains having *rtxBDC* at one locus and *rtxCAtolC* at a separate locus, resulting in two copies of *rtxC* and one copy of the other four genes. Other strains have all five *rtxBDCAtolC* genes at one locus and *rtxCAtolC* at the other locus, resulting in two copies of *rtxC*, *rtxA*, and *tolC* [14]. The functional consequences of these different gene arrangements are currently unknown.

RtxA functions as a hemolysin and cytotoxin by forming pores in the host cell membrane and was demonstrated to form 1.9 nm pores in lipid bilayers in vitro [15]. The process of pore formation requires RtxC-mediated acylation of RtxA, specifically with 14-carbon fatty acyl chains [16], and pore formation is enhanced by binding of RtxA to cholesterol [17]. Unlike some other members of the RTX family of cytotoxins, RtxA does not require β_2_ integrins for membrane insertion but does depend on cell surface oligosaccharides for optimal activity [18]. In addition to being freely secreted, RtxA was also demonstrated to associate with outer membrane vesicles (OMVs), and these OMVs could efficiently deliver toxins to host cells [19].

### 3.2. Role of Type IV Pili in Invasion

There are multiple reports that antecedent or concurrent upper respiratory viral infection is associated with cases of invasive *K. kingae* disease [20,21,22,23], suggesting that viral-induced damage, in addition to RtxA-mediated damage, to the oropharyngeal epithelium may play a role in *K. kingae* bloodstream invasion. While there is currently no known direct link between type IV pili and invasion, it was noted that *K. kingae* isolates collected from the upper respiratory tract were more likely to be piliated than strains isolated from the blood or joint fluid [4]. Interestingly, the PilC1 pilus-associated adhesin discussed above was shown to mediate adherence to the extracellular matrix (ECM) components laminin and collagen IV, which are found in the basement membrane below epithelial cells, as well as collagen I, which composes the interstitial connective tissue layer below the basement membrane [7]. These ECM components can become exposed following damage to the epithelium, potentially providing a route for *K. kingae* to invade and access the vasculature.

## 4. Survival in the Bloodstream

### 4.1. Polysaccharide Capsule

For *K. kingae* to produce invasive disease, it must survive in the bloodstream to disseminate, typically to the joints, bones, or more rarely the endocardium. The blood is considered a hostile environment for invading microorganisms, and numerous strategies for resisting the innate and adaptive immune pressures present in this environment have evolved. Elaboration of a polysaccharide capsule by numerous Gram-negative bacteria was shown to be critical for bloodstream survival. Investigation into the genetic requirements of encapsulation revealed multiple loci critical for surface presentation of the capsule in *K. kingae*. The *ctrABCD* operon is required for capsule export [9,24]; the *lipA* and *lipB* genes are both essential for surface localization of capsule, and homologs of these genes in *Escherichia coli* and *N. meningitidis* were shown to assemble the β-Kdo linker between the lipid membrane anchor and the capsular polysaccharide [24,25]; and the *csaA* gene product (encoded in the region called the capsule synthesis locus) is the capsule synthase required for polymerizing the GalNAc-Kdo capsule polymer of prototype strain KK01 [24,26,27]. Site-directed mutagenesis studies targeting predicted active site residues in CsaA identified this protein as a bifunctional glycosyltransferase responsible for catalyzing both linkages in the GalNAc-Kdo capsule polymer.

Investigation of encapsulation in the *K. kingae* population revealed that there are four capsular polysaccharides represented in a diverse collection of clinical isolates that are termed types a, b, c, and d [26,27,28]. The type a capsule is a polymer of [3)-β-Gal*p*NAc-(1→5)-β-Kdo*p*-(2→], the type b capsule is a polymer of [6)-α-D-Glc*p*NAc-(1→5)-β-(8-OAc)Kdo*p*-(2→], the type capsule is a polymer of [3)-β-D-Rib*f*-(1→2)-β-D-Rib*f*-(1→2)-β-D-Rib*f*-(1→4)-β-Kdo*p*-(2→], and the type d capsule is a polymer of [P-(O→3)[β-D-Gal*p*-(1→4)]-β-D-Glc*p*NAc-(1→3)-α-D-Glc*p*NAc-1-] [26,27,28]. Of note, the genomic location of the capsule synthesis locus is maintained across the *K. kingae* population structure, but the gene content varies depending on the capsule type [26]. By expressing the different capsule synthesis loci (termed *csa*, *csb*, *csc*, and *csd*) in an isogenic background, it was empirically demonstrated that the contents of this locus define the polysaccharide type in *K. kingae* [26]. Interestingly, there is a difference in capsule type representation in strains collected from healthy carriers versus strains collected from patients with invasive *K. kingae* disease. While type c and type d capsules combined make up ~30% of healthy carrier isolates, they only make up <5% of invasive disease isolates, suggesting that strains that express the type a and type b capsules have greater pathogenic potential [26,29,30].

Initial studies examining interactions of *K. kingae* with normal human serum revealed high-level resistance to complement-mediated lysis [31]. Capsules and an additional extracellular polysaccharide called the galactan exopolysaccharide (discussed in more detail below) were found to both enhance survival in human serum through prevention of the complement factor and antibody opsonization on the bacterial surface [31]. In addition, mutants lacking a capsule displayed an intermediate virulence phenotype in the juvenile rat infection model [31]. Studies of in vitro interactions between *K. kingae* and human neutrophils revealed that the capsule plays a key role in preventing bacteria–neutrophil interactions and preventing neutrophil-mediated killing [32]. The capsule was specifically implicated in dampening the ability of neutrophils to generate reactive oxygen species [32].

### 4.2. Galactan Exopolysaccharide

Bendaoud et al. found that *K. kingae* extracts contained a substance that displayed broad-spectrum anti-biofilm activity [28]. Chemical and structural analyses revealed that this material, collected from the clinical isolate PYKK181, was a homopolymer of galactofuranose with the structure of [▯→3)-β-Gal*f*-(1→6)-β-Gal*f*-(1→]_n_. Synthesis of this polysaccharide was dependent on the *pamA*, *pamB,* and *pamC* genes, as expression of these three genes in *E. coli* resulted in production of the galactan [28]. Analysis of strain KK01 reveal a similar galactan with a different linkage connecting the galactofuranose residues: [→5)-β-Gal*f*-(1→]_n_ [27]. These are the only two documented galactan structures in *K. kingae*, and additional analyses are necessary to determine how widespread galactan production is and the diversity of galactan structures in this species.

Like the polysaccharide capsule, the galactan has demonstrated roles in immune evasion [31,32]. Specifically, the galactan can independently prevent complement factor and antibody deposition on the bacterial surface and promote serum survival [31]. Also like the capsule, mutants lacking the galactan were attenuated but not completely avirulent in the juvenile rat infection model [31]. The role of the galactan in interactions with neutrophils is distinct from the capsule; it plays a role in preventing neutrophils phagocytosis and has an additional role in resisting the bactericidal effects of antimicrobial peptides [32]. Given these functions, it was suggested that the galactan associates with the bacterial surface through an unknown mechanism [27,31,32], and this is an active area of investigation.

## 5. Invasion of the Joints and Bones

The factors and mechanisms utilized by *K. kingae* to extricate itself from the vasculature to seed the sites of invasive disease, primarily the joints and bones, are largely unexplored. There are currently no published studies examining *K. kingae* adherence to endothelial cells or mechanisms of endothelial breach. Due to the limitations of the juvenile rat infection model, a better animal model that more closely resembles natural human infection will be critical for understanding this step in the pathogenesis of *K. kingae* disease.

How the above-described virulence factors, as well as unknown virulence factors, are regulated and how they interact at the different stages of infection is also largely unknown. Piliation levels vary greatly in the *K. kingae* population [4], and strains isolated from sites of invasive disease are less likely to be piliated than respiratory isolates, suggesting that piliation is downregulated or selected against as the bacteria establish focal infections at normally sterile body sites [4]. *K. kingae* possesses a phase-variable type III methylation system that modifies the expression of numerous genes through action of the ModK methyltransferase [33]. Included in this regulon is the *rtxA* gene(s), indicating that toxin expression is carefully regulated, albeit by unknown environmental signals. Future studies are necessary to uncover both the mechanisms of joint and bone invasion and the regulatory signals that govern this tissue tropism.

## 6. Conclusions

Many insights into the virulence factor repertoire of *K. kingae* were gleaned over the past two decades, and this information has the potential to inform strategies to prevent *K. kingae* disease. Approaches to prevent *K. kingae* adherence to the respiratory epithelium through disruption of type IV pili and/or Knh could be a viable strategy to prevent colonization. As polysaccharide capsules in multiple bacterial pathogens, including *Haemophilus influenzae* type b, *Streptococcus pneumoniae*, and *N. meningitidis*, were successfully incorporated into highly effective glycoconjugate vaccines, it is interesting to speculate that this approach could be applied to *K. kingae*. As only two capsule types (types a and b) make up >95% of invasive disease isolates in all collections examined to date [26,29,30], a glycoconjugate vaccine containing these two polysaccharides could potentially prevent the vast majority of invasive *K. kingae* disease.
